# Promoter methylation of IGFBP-3 and p53 expression in ovarian endometrioid carcinoma

**DOI:** 10.1186/1476-4598-8-120

**Published:** 2009-12-11

**Authors:** Pao-Ling Torng, Ching-Wei Lin, Michael WY Chan, Hui-Wen Yang, Su-Cheng Huang, Chin-Tarng Lin

**Affiliations:** 1 Department of Obstetric and Gynecology, National Taiwan University Hospital and National Taiwan University College of Medicine, Taipei, Taiwan; 2 Institute of Pathology, National Taiwan University College of Medicine, Taipei, Taiwan; 3 Department of Life Science and Institute of Molecular Biology, National Chung Cheng University, Min-Hsiung, Chia-Yi, Taiwan; 4 Department of Pathology, National Taiwan University Hospital, Taipei, Taiwan

## Abstract

**Background:**

Insulin-like growth factor binding protein (IGFBP-3) is an antiproliferative, pro-apoptotic and invasion suppressor protein which is transcriptionally regulated by p53. Promoter methylation has been linked to gene silencing and cancer progression. We studied the correlation between IGFBP-3 and p53 expression as well as *IGFBP-3 *promoter methylation in ovarian endometrioid carcinoma (OEC) by immunohistochemical staining and quantitative methylation-specific PCR (qMSP). Additionally, we assessed the molecular regulatory mechanism of wild type (wt) p53 on IGFBP-3 expression using two subclones of OEC, the OVTW59-P0 (low invasive) and P4 (high invasive) sublines.

**Results:**

In 60 cases of OEC, 40.0% showed lower IGFBP-3 expression which was significantly correlated with higher *IGFBP-3 *promoter methylation. p53 overexpression was detected in 35.0% of OEC and was unrelated to clinical outcomes and IGFBP-3. By Kaplan-Meier analysis, patients with lower IGFBP-3, higher *IGFBP-3 *promoter methylation, and normal p53 were associated most significantly with lower survival rates. In OEC cell line, IGFBP-3 expression was correlated with *IGFBP-3 *promoter methylation. IGFBP-3 expression was restored after treatment with a DNA methy-transferase inhibitors (5-aza-deoxycytidine) and suppressed by a p53 inhibitor (pifithrin-α). The putative p53 regulatory sites on the promoter of *IGFBP-3 *were identified at -210, -206, -183 and -179 bases upstream of the transcription start site. Directed mutagenesis at these sites quantitatively reduced the transcription activity of IGFBP-3.

**Conclusion:**

Our data suggests that IGFBP-3 silencing through *IGFBP-3 *promoter methylation in the absence of p53 overexpression is associated with cancer progression. These results support a potential role of *IGFBP-3 *methylation in the carcinogenesis of OEC.

## Background

Epithelial ovarian cancer is the most lethal gynecological cancer among women worldwide. Metastasis occurs in most patients before diagnosis, and the majority of patients have high subsequent recurrent rate after completing surgery and chemotherapy [1]. Survival is at about 3 years for patients in advanced stages [2]. The poor prognosis of ovarian cancer is due to the difficulty in early diagnosis and the detrimental processes of invasion and metastasis. A better understanding of the molecular mechanisms of cancer development and progression will help to improve the diagnosis and treatment of the disease.

Insulin-like growth factor binding proteins (IGFBPs) are circulating transport proteins for IGF, with IGFBP-3 being the predominant IGFBP in circulation [3]. IGFBP-3 can regulate cell growth and death, either dependent or independent of its interaction with IGF [3,4]. Recently, we have further identified *IGFBP-3 *as an invasion suppressor gene using an established ovarian cancer cell line OVTW59-P0 and its sublines P1 to P4, which were obtained from a gradual invasion model with sequential increase in invasiveness [5]. Many tumor suppressor genes involved in cancer formation and progression are frequently epigenetically silenced through aberrant hypermethylation at their promoter regions [6]. In ovarian cancer, genes with promoter hypermethylation are frequently found to be related to cancer progression [7,8]. Aberrant promoter hypermethylation of *IGFBP-3 *and gene silencing are observed in many cancers, such as lung, hepatocellular, gastric, colorectal, breast, and ovarian cancers [9-13]. However, the association of *IGFBP-3 *promoter hypermethylation with poor clinical outcome was identified only at early stages in lung and ovarian cancers [9,10].

p53 is a known transcription factor for IGFBP-3 expression [9]. Induction of IGFBP-3 by p53 has been shown to cause cell apoptosis in an IGF-independent manner [14]. Eleven p53 binding sites have been identified within IGFBP-3 gene based on the homology to the p53 binding consensus sequence, and confirmation by electrophoretic mobility shift analyses [15]. Promoter hypermethylation at these p53 binding sites caused gene silencing and resistance to p53 [16]. Therefore, it has been suggested that p53 could mediate cross-talk to the IGF axis through IGFBP-3 regulation [14].

Pathologically, epithelial ovarian cancer is classified into four major histological subtypes: serous, mucinous, endometrioid and clear cell carcinoma. Each subtype is associated with distinct molecular alterations [17]. In our previous study, we found 51.4% of the ovarian endometrioid carcinoma (OEC) subtype showing lower IGFBP-3 expression, which is associated significantly with poor patient outcome [5]. From literature review, p53 overexpression is more frequently reported in the serous subtype of ovarian cancer [18-20]. The association of altered p53 expression in tumor tissue with patient survival in ovarian cancer is still under debate [19-21], but a significant correlation is often reported in the serous subtype [18,19]. Despite the close molecular regulation between p53 and IGFBP-3 being known for more than a decade [9], little information concerning p53 regulation of IGFBP-3 or the effect of epigenetic inactivation of IGFBP-3 exist with defined clinical endpoints. In the current study, we analyzed the association between p53 and IGFBP-3 expression in ovarian cancer progression. We explored the clinical significance of aberrant promoter hypermethylation at the p53 binding sites of *IGFBP-3 *in OEC. Functional analysis of p53 regulation on IGFBP-3 expression was further assessed using the OVTW59-P0 and P4 cell lines. Our study indicates that methylation at the p53 transcription factor binding sites in the *IGFBP-3 *promoter can silence IGFBP-3 expression and hence lead to cancer progression in OEC.

## Results

### IGFBP-3 expression was correlated with IGFBP-3 promoter methylation, but not p53 expression

**Figure 1:**
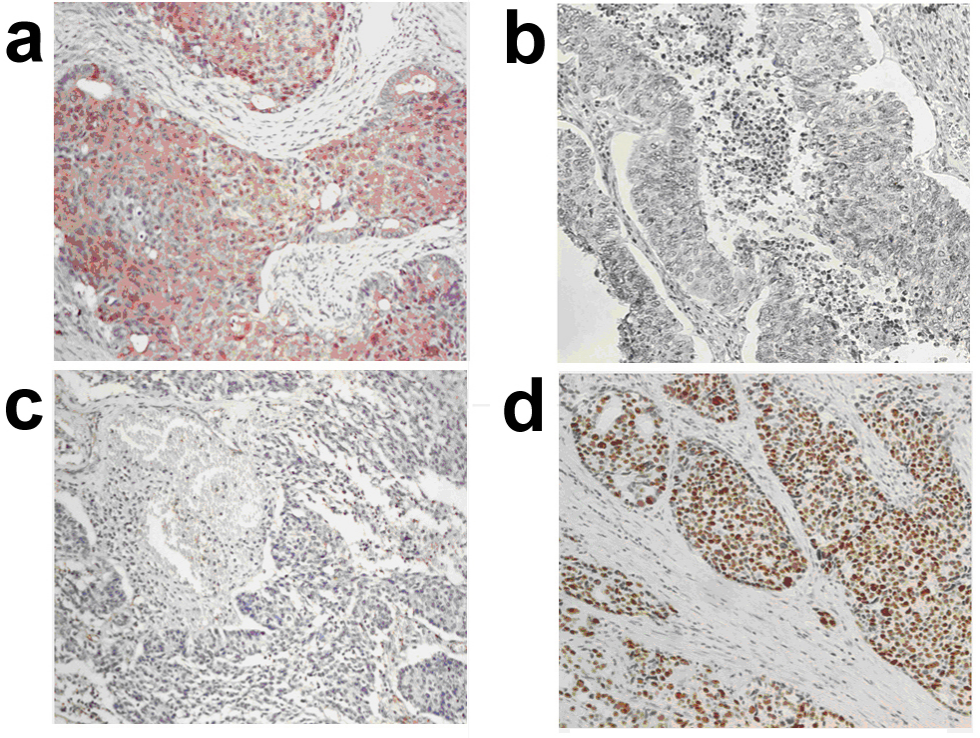
**Immunohistochemical staining**. High IGFBP-3 expression and normal p53 in a case of grade 1 OEC (a and b); and low IGFBP-3 expression and overexpression of p53 in a case of grade 3 OEC (c and d) (a - d, H&E, × 200).

A total of 60 OEC patients with an average age at surgery of 51.5 ± 12.4 years old were recruited. Among these patients, 41.7% were in the advanced disease stages (stages 3 and 4) and 61.8% at higher histological grades (grades 2 and 3) (Table 1). By immunohistochemical staining, IGFBP-3 was located in the tumor cytoplasm and p53 was immunoreactive at the nucleus (Figure 1).

**Table 1:** Tumor expressions of IGFBP-3, p53 and *IGFBP-3 *promoter methylation and the clinicopathological features in patients with OEC.

Characteristics	IGFBP-3			p53			*IGFBP-3 *Methylation		
			
	High N = 36	Low N = 24	*p *value	Normal N = 39	Overexpression N = 21	*p-*value	≤ 3% N = 20	> 3% N = 20	*p *value
Age (mean ± SD)	51.7 ± 11.9	52.2 ± 12.7	0.88	49.3 ± 12.3	55.7 ± 11.7	0.06	51.8 ± 12.9	51.5 ± 13.3	0.94
Stage									
I	22	6		20	8		11	8	
II	5	2	0.02	5	2	0.54	4	1	0.24
III	9	15		14	10		5	10	
IV	0	1		0	1		0	1	
Grade									
1	19	0		16	3		11	4	
2	11	12	< 0.001	12	11	0.09	5	9	0.05
3	6	11		11	6		3	7	
Low IGFBP-3				17 (43.6%)	7 (33.3%)	0.77	2 (10.0%)	12 (60.0%)	0.003
p53 overexpression	14 (38.9%)	7 (29.2%)	0.62				8 (12.5%)	6 (30.0%)	0.11

The amount of methylation at *IGFBP-3 *promoter in these patient samples was measured by quantitative real time methylation specific PCR (qMSP). Twenty samples were unable to be bisulfite converted probably due to failed in DNA extraction from very old paraffin blocks. In total, qMSP analysis was performed on 40 patient samples. There were no differences in IGFBP-3 and p53 expressions or in clinicopathological features between the samples which were unable to be analyzed by qMSP and those which were (Additional file 1, Table S1). Among these 40 samples, the calculated promoter methylation of *IGFBP-3 *ranged from 0.01 to 59.8%. By using receiver operating characteristic (ROC) curve and area under the curve (AUC) analysis methods, the optimal cutoff value was set at a threshold of > 3% as hypermethylation. At this threshold, the sensitivity of *IGFBP-3 *methylation with low IGFBP-3 expression was 0.86 and the specificity was 0.73 (Additional file 1, Figure S1).

Table 1 shows the correlation among IGFBP-3 and p53 expression and qMSP results with the clinical parameters. High IGFBP-3 expression was found in tumors of lower grades and lower stages, and p53 tended to be overexpressed in tumors with higher grades. No correlation was found between p53 overexpression and IGFBP-3 expression or *IGFBP-3 *promoter methylation. Cases that showed higher *IGFBP-3 *methylation (at threshold > 3%) were significantly correlated with lower IGFBP-3 expression and higher histological grades.

The calculated *IGFBP-3 *methylation from qMSP analysis was further subdivided into four quartiles, and Table 2 shows the relation between IGFBP-3 expression and clinicopathological features for each quartile. Again, we observed a close association of high IGFBP-3 expression with ≤ 3% methylation and low IGFBP-3 expression with > 3% methylation. However, 2 cases showed low IGFBP-3 expression and ≤ 3% methylation and 8 cases showed high IGFBP-3 expression and > 3% methylation. These associations were opposite to the parallel relationship of methylation and IGFBP-3 suppression and were thus grouped into the unparallel category (Table 3).

**Table 2:** Relation among the four quartiles of *IGFBP-3 *promoter methylation by qMSP analysis with IGFBP-3 expression and clinicopathological features in OEC patients.

Quartiles	***IGFBP-3 *methylation** (% range)	Number of patients (%)				
		
		Low IGFBP-3	Advanced stage	Higher grade	Patients with recurrent tumor	Patients expired
First	0.01 -- 1.07	1 (10)	4 (40)	4 (40)	4 (40)	2 (20)
Second	1.12 -- 2.83	1 (10)	1 (10)	5 (50)	2 (20)	2 (20)
Third	3.10 -- 6.36	5 (50)	5 (50)	9 (90)	5 (50)	5 (50)
fourth	6.88 -- 59.79	7 (70)	6 (60)	7 (70)	5 (50)	4 (40)

Advanced stage was defined as stage 3 or 4.

Higher histological grade was defined as grade 2 or 3.

**Table 3:** Survival Analysis for patients with OEC.

Variables	Case number	PFS						OS					
		Unadjusted			Adjusted*			Unadjusted			Adjusted*		
					
		HR	95% CI	*p *value	HR	95% CI	*p *value	HR	95% CI	*p *value	HR	95% CI	*p *value
**IGFBP-3**													
High	36	1.00	1.36-7.77	0.01	1.00	0.27-2.27	0.65	1.00	1.28-9.38	0.01	1.00	0.27-2.85	0.82
Low	24	3.25			0.78			3.46			0.87		
**P53**													
Normal	39	1.00	0.43-2.42	0.97	1.00	0.53-3.74	0.49	1.00	0.29-2.02	0.60	1.00	0.24-2.32	0.62
Overexpression	21	1.01			1.41			0.77			0.75		
**Methylation**													
≤ 3%	20	1.00	0.76-5.82	0.14	1.00	0.20-2.033	0.45	1.00	0.91-9.60	0.07	1.00	0.16-2.45	0.49
> 3%	20	2.11			0.64			2.95			0.62		
**IGFBP-3 expression and *IGFBP-3 *methylation**													
High IGFBP-3 Methylation ≤ 3%	18	1.00			1.00			1.00			1.00		
Unparallel	10	2.38	0.53-10.64	0.26	1.46	0.09-2.49	0.37	3.16	0.53-19.01	0.21	0.56	0.08-3.78	0.56
Low IGFBP-3 Methylation > 3%	12	4.45	1.17-16.85	0.03	0.37	0.07-1.88	0.23	7.18	1.48-34.89	0.02	0.64	0.11-3.68	0.62
**IGFBP-3 expression and *IGFBP-3 *methylation with normal p53**													
High IGFBP-3 Methylation ≤ 3%	10	1.00			1.00			1.00			1.00		
Unparallel	6	4.67	0.58-37.40	0.15	0.47	0.03-7.09	0.56	4.81	0.57-40.40	0.15	0.37	0.18-7.68	0.52
Low IGFBP-3 Methylation > 3%	8	21.47	2.57-179.19	0.005	0.40	0.07-28.05	0.82	20.38	2.39-173.89	0.006	0.79	0.27-23.40	0.89

PFS indicates progression-free survival; OS indicates overall survival; HR indicates hazard ratio; 95% CI confidence interval; IGFBP-3, insulin-like growth factor binding protein-3; Unparallel indicates either high IGFBP-3 and *IGFBP-3 *promoter methylation > 3% or low IGFBP-3 and *IGFBP-3 *promoter methylation ≤ 3%.

*Adjusted by age at diagnosis, disease stage and histological grade.

### Clinical correlation of IGFBP-3 expression, p53 overexpression and IGFBP-3 promoter methylation with survival rates

Cox-regression analysis revealed low IGFBP-3 expression to be associated with lower progression-free survival (PFS) and lower overall survival (OS) rates. p53 overexpression was not associated with patient survivals while high *IGFBP-3 *promoter methylation was marginally associated with lower OS. When combining these variables for survival analysis, low IGFBP-3 expression, high *IGFBP-3 *promoter methylation and normal p53 expression were correlated most significantly with lower PFS and OS. However, since IGFBP-3 expression and *IGFBP-3 *promoter methylation were correlated with disease stages and tumor grades (Table 1), the associations of these variables with survival rates was not independent of age, stage and grade (Table 3).

**Figure 2:**
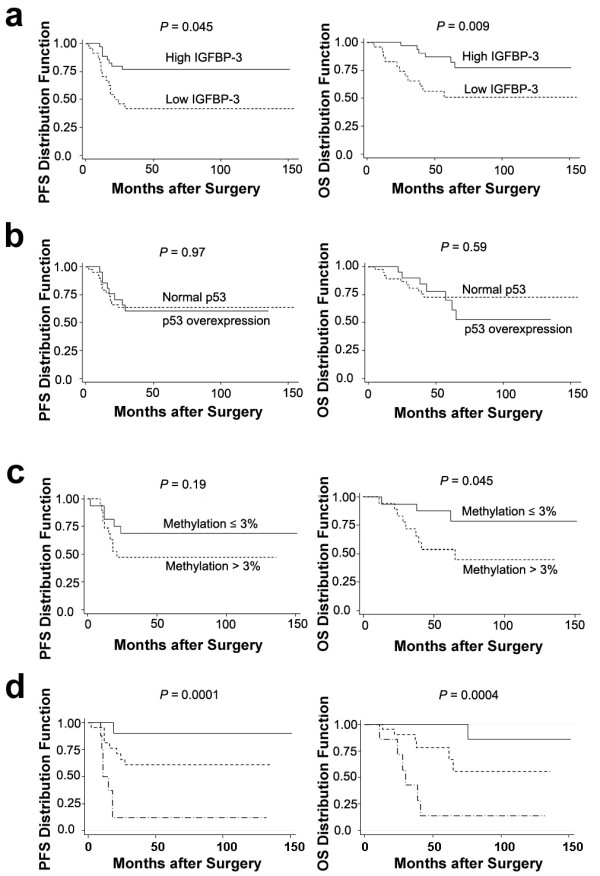
**Kaplan Meier survival estimate of progression-free survival (PFS) and overall survival (OS) for OEC patients classified by (a) IGFBP-3 expression, (b) *IGFBP-3 *promoter methylation status and (c) p53 expression. (d) Survival estimate for patients with normal p53, classified according to IGFBP-3 expressions and *IGFBP-3 *promoter methylation status**. --- indicates patients with high IGFBP-3 and *IGFBP-3 *promoter methylation ≤ 3%; --- indicates patients with unparallel IGFBP-3 and *IGFBP-3 *promoter methylation, i.e. either high IGFBP-3 and *IGFBP-3 *promoter methylation >; 3% or low IGFBP-3 and *IGFBP-3 *promoter methylation ≤ 3%; --·-indicates patients with low IGFBP-3 and *IGFBP-3 *promoter methylation > 3%. Logrank test was used to compare survival distributions.

Figure 2 shows Kaplan Meier survival estimates of patients classified by IGFBP-3 expression, methylation levels of *IGFBP-3 *promoter and p53 expression. For comparison, Additional file 1, Figure S2 shows the survival estimates classified by IGFBP-3 expression and *IGFBP-3 *promoter methylation levels in all patients with tumors that showed either normal or overexpressed p53. The association of patient PFS and OS with IGFBP-3 expression and *IGFBP-3 *promoter methylation were less significant comparing to the results when only cases with normal p53 were included (Figure 2d).

### Correlation of IGFBP-3 promoter methylation and IGFBP-3 expression in ovarian cancer cell lines

**Figure 3:**
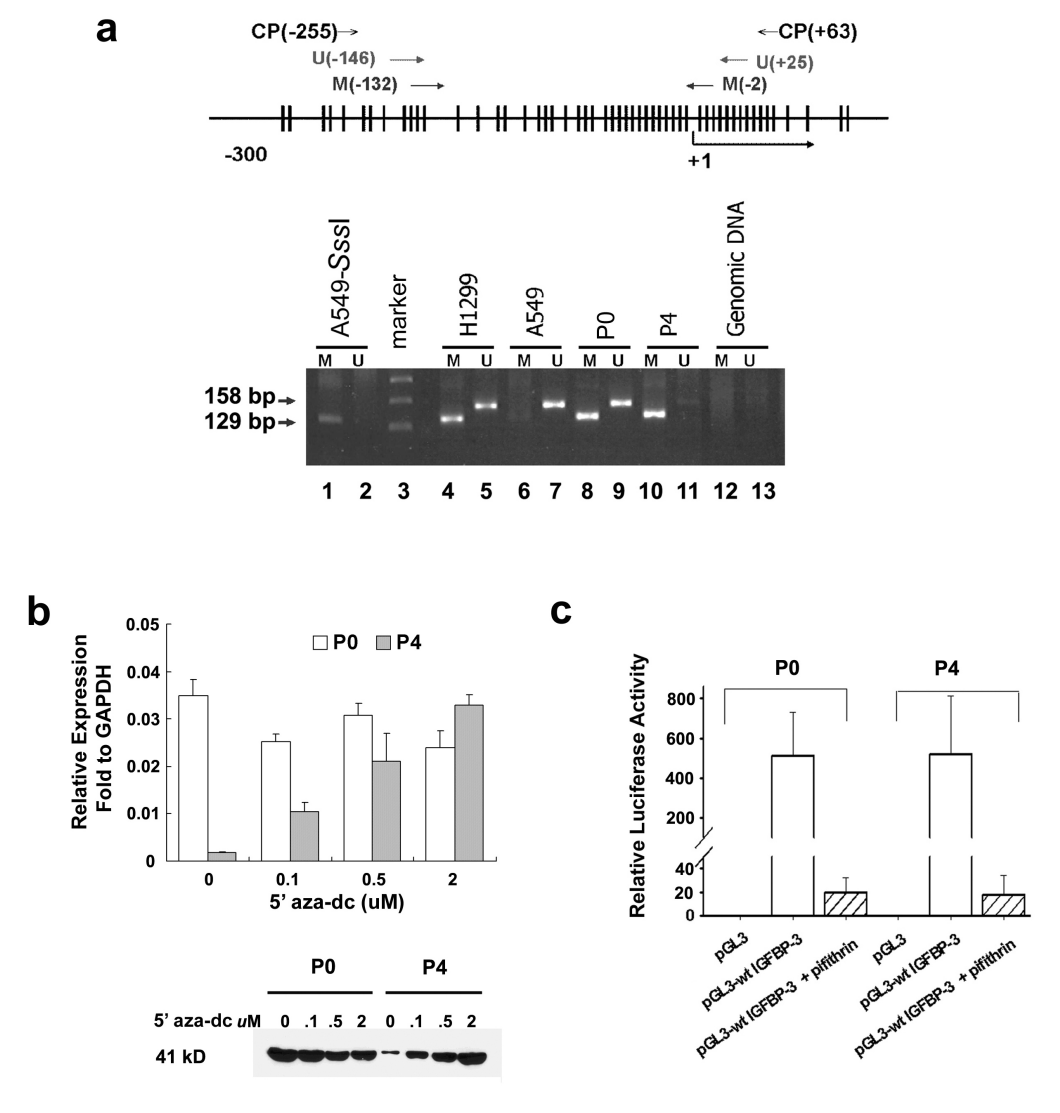
**Aberrant promoter methylation and transcriptional regulation of p53 at *IGFBP-3***. (a) MSP shows *IGFBP-3 *promoter methylation in OEC cell lines. Upper panel: schematic diagram showing CpG islands in the IGFBP-3 promoter and the location of primers used (M, methylated; U, unmethylated). Arabic numbers indicate the location of nucleotides relative to the transcription start site (TSS) (+1). Vertical lines represent the position of the CpG dinucleotides. Lower panel: MSP in P0 and P4 sublines. U indicates unmethylated (158 bp) and M indicates methylated (129 bp) PCR products. The *Sss*I treated A549 (lanes 1 and 2) and H1299 (lanes 4 and 5) were used as methylated controls, A549 as unmethylated control (lanes 6 and 7), and Genomic DNA without bisulfite conversion as negative control (lanes 12 and 13). (b) Real-time PCR analysis of IGFBP-3 mRNA showing restoration of IGFBP-3 expression in P4. P0 and P4 cell lines were treated with 0, 0.1, 0.5 or 2 μM of 5-aza-dC for 8 days. Culture media was changed to serum free media for 24 hours before IGFBP-3 analysis. Lower panel shows Western blotting corresponding to IGFBP-3 expressions in P0 and P4 culture media after 5-aza-dC treatment. Similar amount of culture media from equal number of cells were loaded in each lane. (c) Luciferase activity after transfection with wild type IGFBP-3 promoter (-253 ~+61)-pGL3 luciferase reporter construct (pGL3-wt IGFBP-3). Luciferase activity was normalized against renilla activity. Transfection with pGL3-basic vector (pGL3) was used as negative control. Transfections were carried out in triplicate and were done in three independent experiments.

To have a better understanding of the molecular regulation of IGFBP-3, we included two established OEC sublines OVTW59-P0 and P4 for further study. As reported previously, P4 was more invasive and metastatic than P0; and P4 expressed very low IGFBP-3 compared to P0 [5]. Using MSP, we detected both 129 bp methylated and 158 unmethylated bands in P0 indicating that P0 was partially methylated at the *IGFBP-3 *promoter sites. P4 showed only a 129 bp band indicating a complete methylation of the *IGFBP-3 *promoter (Figure 3a). 5-aza-deoxycytidine (5-aza-dC) treatment restored IGFBP-3 expression in P4. The amount of 5-aza-dC used for treatment directly correlated with IGFBP-3 mRNA and protein expressions (Figure 3b).

### Transcriptional regulation of p53 on IGFBP-3 promoter

Both P0 and P4 expressed similar levels of wild type (wt) p53 by direct sequencing after amplifying exons 5, 6, 7, and 8 of *p53 *gene, and Western blot analysis (data not shown). To test the transcriptional regulation of *IGFBP-3 *by p53, the -253 ~+61 portion of the promoter and first exon of *IGFBP-3 *were transfected into P0 and P4. After treatment with a known p53 inhibitor, pifithrin-α, the luciferase activity representing IGFBP-3 expression was completely abolished in these two sublines (Figure 3c). This result indicates that promoter sites between -253 ~+61 are essential for transcriptional regulation by p53 on IGFBP-3 expression

### Identification of methylation hotspots at the p53 consensusbinding sequence in IGFBP-3 promoter

**Figure 4:**
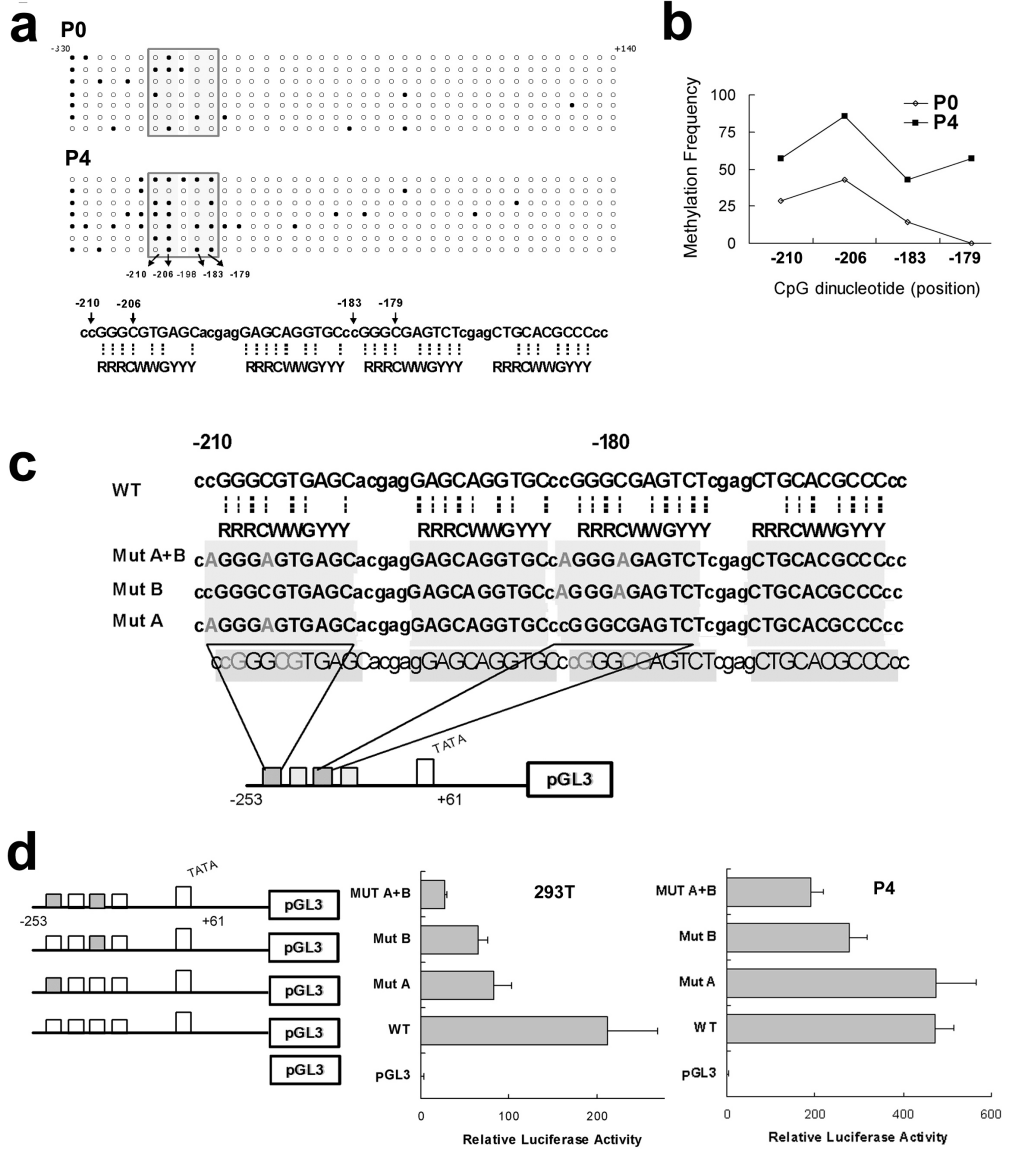
**Identification of methylation sites at p53 binding sequence of IGFBP-3 promoter**. (a) BSP in P0 and P4. Forty CpG sites were shown and each circle represents a CpG dinucleotide. Open circle represents non-methylated CpG dinucleotude and black circle represents methylated CpG dinucleotide. The enclosed boxes represent CpG dinucleotide located in the p53 consensus binding site. Seven clones were sequenced in each cell line. (b) The methylation frequency in P0 and P4 from -210 to -179 regions of IGFBP-3 promoter. (c) Schematic diagram of designed constructs for site-directed mutagenesis assay. The wild type construct contains IGFBP-3 promoter region from --253 ~+61. The mutant sequences carry the same region but with point mutations: (-210, -206) represent as Mut A constructs, and (-183, -179) represent as Mut B constructs. Mut A+B construct contains all four nucleotide mutation. (d) Luciferase activities of 293T and P4 transfected with wild/mutant types of IGFBP-3 promoter constructs. Luciferase activity was normalized against renilla activity. Transfection with pGL3-basic vector (pGL3) was used as negative control. Transfections were carried out in triplicate and were done in at least three independent experiments.

Bisulfite-PCR and sequencing (BSP) was performed in P0 and P4, focusing on the -330 to +140 sites of *IGFBP-3 *that included the reported 11 p53 consensus binding sites [22]. We identified four CpG sites that were frequently methylated in these two sublines, distributed at the -210, -206, -183 and -179 loci of *IGFBP-3 *promoter (Figure 4a). P4 showed a higher frequency of methylation at these loci compared to P0 (60.7% versus 25.0%) (Figure 4b).

Since p53 binding at these sequences has been reported to be diminished by methylation [16], we established a series of site-directed mutation constructs to test the importance of these four loci on the transcriptional regulation by p53 of *IGFBP-3 *(Figure 4c). In addition to using P0 and P4, a human embryonic 293T kidney cell line with documented wt *p53 *was also included in analysis. The transcription activity of *IGFBP-3 *was significantly reduced after mutagenesis at these loci and the amount of luciferase activity corresponding to IGFBP-3 expression decreased as the number of mutated loci increased (Figure 4d). These findings indicate that these four loci are critical spots for transcriptional regulation by p53 of *IGFBP-3*. In addition, methylation at these sites could quantitatively silence the process of IGFBP-3 induction by p53.

## Discussion

We demonstrated previously that IGFBP-3 is an invasion suppressor in OEC [5]. In this study, we evaluated the molecular mechanism of IGFBP-3 regulation in cancer cells and found that methylation at the CpG sites of the *IGFBP-3 *promoter was significantly correlated with IGFBP-3 silencing in OEC tumors. Clinically, patients with lower IGFBP-3 expression and higher *IGFBP-3 *promoter methylation have significantly lower PFS and OS. This association was strongest in cases with normal p53 expression. In an *in vitro *study using OEC cell line OVTW59, we identified a quantitative correlation between IGFBP-3 suppression and methylation in the first 200 base pairs surrounding the transcription start site of *IGFBP-3*. Our data suggest that IGFBP-3 silencing through aberrant hypermethylation at its promoter is a major regulatory pathway for OEC tumorigenesis.

Methylation specific PCR (MSP) has been widely used to study promoter methylation in clinical specimens. By MSP, aberrant methylation at the *IGFBP-3 *promoter was found in 44% of epithelial ovarian cancer and in 28.6% (12/42) of OEC subtype [7,12]. However, IGFBP-3 silencing has been reported unrelated to *IGFBP-3 *promoter methylation [7,12]. A significant association between *IGFBP-3 *promoter methylation and disease progression has been reported, but only in early-stage ovarian cancer [12]. It was concluded that methylation at *IGFBP-3 *promoter is a common tumorigenesis process in the early steps of ovarian cancer progression [12]. By setting the calculated percentage of IGFBP-3 methylation at 3% as a cutoff point for qMSP analysis, we found methylation at the *IGFBP-3 *promoter significantly correlated with IGFBP-3 silencing in OEC. Furthermore, we found patients with lower IGFBP-3 expression and higher *IGFBP-3 *promoter methylation to be significantly associated with poor outcomes. A possible reason is that we have used a more sensitive method, qMSP, to study these epigenetic events. In addition, we have focused our study on the OEC subtype of ovarian cancer. It has been observed that promoter methylation of specific genes in cancer occur frequently in a tumor-type and cell-type specific manner [23]. Our results demonstrate that the clinical significance of aberrant *IGFBP-3 *promoter methylation is more commonly observed in the OEC subtype of ovarian cancer.

Ten cases in our study showed unparallel results between IGFBP-3 expression and *IGFBP-3 *promoter methylation, i.e. either high IGFBP-3 and *IGFBP-3 *promoter methylation > 3% or low IGFBP-3 and *IGFBP-3 *promoter methylation ≤ 3%. Though threshold of > 3% as hypermethylation was selected based on ROC curve analysis that showed 0.86 sensitivity and 0.73 specificity of low IGFBP-3 expression, we found five (50%) of these unparallel cases were at the third quartile of methylation, i.e. high IGFBP-3 expression and promoter methylation levels between 3% and 6.36% (Table 2). This could be due to inadequate sensitivity and specificity of qMSP assay. Alternatively, the unparallel result could be explained by multiple mechanisms of IGFBP-3 regulation. IGFBP-3 could also interact with several other growth-inhibitory agents to mediate wide varieties of growth suppression signal in the absence of IGF [24,25]. In addition, other CpG methylation sites in the *IGFBP-3 *promoter that were not studied might also contribute to IGFBP-3 silencing

We further explored the association between p53 and *IGFBP-3 *promoter methylation and the mechanism of p53 regulation on IGFBP-3. Thirty-five percent of these cases showed p53 overexpression that was correlated with higher tumor grade at marginal significance. However, overexpression of p53 was not correlated with IGFBP-3 expression or *IGFBP-3 *promoter methylation and it did not correlate with OEC patient survival. This suggested that p53 is not important in the progression of OEC, and that IGFBP-3 silencing through promoter methylation is a mechanism different from p53 overexpression. Since OEC is similar in histological pattern to the endometrioid (EC) subtype of endometrial cancer, we reviewed the molecular characteristics of different subtypes of endometrial cancer. In endometrial cancer, p53 alteration was specifically reported to be present in the non-endometrioid (non-EC) serous subtype, which has been classified as a type 2 endometrial cancer. The EC subtype of endometrial cancer is classified as type 1 endometrial cancer [26]. Accordingly, Kurman *et al*. proposed an epithelial ovarian cancer model composed of type 1 and type 2 tumors based on: the histological patterns, molecular features, and the process of tumor progression. Type 1 tumors are slow growing, genetically stable, and are characterized by mutations in KRAS, BRAF, PTEN, and beta-catenin. Type 2 tumors are highly aggressive, rapidly growing, genetically instable, and are characterized by mutation of TP53 [20]. Based on this model, the OEC subtype is classified as a type 1 tumor and serous subtype, with characteristic TP53 mutations, is classified as a type 2 tumor [20]. In our study, we confirmed that p53 alteration is not a characteristic of OEC. Instead, low IGFBP-3 and high *IGFBP-3 *promoter methylation are major prognostic factors for OEC. By survival analysis, patient survival is lowest in cases with low IGFBP-3, high *IGFBP-3 *promoter methylation, and normal p53. These suggest OEC is a distinct subtype of ovarian cancer that IGFBP-3 silencing through *IGFBP-3 *promoter methylation could play an important role in cancer development and progression. We found p53 alteration is not important for the tumorigenesis of OEC, but IGFBP-3 silencing through *IGFBP-3 *promoter methylation might subsequently interrupt the communication network between p53 and the IGF axis, and hence lead to cancer progression of OEC.

Of the eleven p53 binding sites in IGFBP-3 gene that has been identified [22], Hanafusa *et al*. observed that at least four sites between -210 to -150 are essential for p53 induced expression of IGFBP-3 in human hepatocarcinoma cell line HepG2. They also found that hypermethylation of these sequences could selectively suppress p53 induced IGFBP-3 expression [16]. Using our OEC cell line, we identified four sites in the *IGFBP-3 *promoter at the -210, -206, -183, and -179 loci, as methylation hot spots. Functional analyses have been performed to study the influence of methylation at these sites on the p53 regulation of IGFBP-3. Hanafusa *et al*. showed diminished p53 binding and IGFBP-3 repression associated with hypermethylation of these sequences by luciferase assay after co-transfection of p53 and mutant IGFBP-3 promoter constructs, and by electrophoresis mobility shift assay [16]. In our study, we used pifithrin-α to inhibit p53 and found a subsequent decrease in IGFBP-3 expression in OVTW59-P4 and 293T cell lines, both of which contain wt p53. These functional analyses support the observation that p53 can up-regulate IGFBP-3 expression [27], and methylation at these sites suppress the p53 activation of IGFBP-3.

Furthermore, our results show a quantitative association between methylation at the *IGFBP-3 *promoter and IGFBP-3 expression. The amount of methylation at these hot spots were positively related to IGFBP-3 suppressions in the P0 and P4 sublines. Using site-directed mutagenesis, we demonstrated a linear correlation between IGFBP-3 suppression and the amount of methylation at these loci. Our *in vitro *data support our clinical qMSP finding that IGFBP-3 expression was correlated to *IGFBP-3 *promoter methylation in OEC tumors.

Our observation that normal p53 is a required factor along with lower IGFBP-3 and higher *IGFBP-3 *promoter methylation for a significant survival result also suggests that wt p53 is an important molecular characteristic of OEC subtype. Recently, Kawasaki *et al*. reported an inverse association between *IGFBP-3 *promoter methylation and microsatellite instability in patients with methylator phenotype colorectal cancers, particularly under the condition of wt p53 [28]. We postulate that the biological environment of *IGFBP-3 *promoter methylation is strictly regulated, such that wt p53 and un-methylated *IGFBP-3 *promoter region are necessary factors to maintain a homeostatic condition.

Progression of tumorigenesis involves multiple steps of genetic alteration. From OEC clinical specimens and cell lines, our data indicates that aberrant methylation at p53 consensus sequence binding region of *IGFBP-3 *promoter could contribute to low IGFBP-3 expression and subsequently to a poor patient survival outcome. Normal p53 plays an important role in this regulation. In conclusion, our studies show clinical evidence on methylation-dependent epigenetic silencing of IGFBP-3 expression regulated by p53. This regulatory pathway represents an important mechanism for loss of IGFBP-3 expression during OEC tumorigenesis and/or progression to metastasis.

## Methods

### Chemical reagents

General laboratory reagents were purchased from Sigma-Aldrich (St. Louis, MO, USA) and cell culture reagents from Gibco/BRL (Grand Island, NY, USA), unless otherwise specified. 5-aza-dC was obtained from Sigma-Aldrich and pifithrin-α from Calbiochem (Gibbstown, NJ, USA).

### Tumor specimen and cell lines

Sixty cases of OEC specimens were collected from the archive of Department of Pathology, National Taiwan University (NTU) Hospital from May, 1994 to September 2006. These patients received surgeries and chemotherapies according to our previous report [29]. Informed consents were obtained for each patient before receiving operations. OEC cell lines OVTW59-P0 and P4 were established in our laboratory previously [5]. Cell lines A549, H1299 and 293T were obtained from Taiwan National Health Research Institute cell bank. All cell lines were maintained in DMEM solution with 5% FCS.

### Immunohistochemical staining

Staining was performed in 4 μm paraffin sections as described previously [29]. In brief, sections were dewaxed, rehydrated, heated by microwave and then blocked with 1% H_2_O_2 _and normal horse serum. Antibodies against IGFBP-3 (Sigma-Aldrich) and p53 (clone DO-7; DAKO, Glostrup, Denmark), as the primary antibodies, were incubated, followed by biotinylated secondary antibodies, avidin-biotin-peroxidase complex and peroxidase substrate were then added for microscopic observation. Interpretations of immunostaining were performed independently by two authors (PLT and CTL). Immunostaining of IGFBP-3 was defined as low or high when less or more, respectively, than 25% of tumor cells exhibited strong staining of cytoplasm. Immunostaining of p53 was defined as normal or overexpressed when less or more, respectively, than 25% of tumor cells exhibited strong nuclear staining.

### Bisulfite modification

Tumor parts from 10 μm paraffin sections were dissected under a stereomicroscope for DNA isolation. An estimation of greater than 80% purity of tumor cells was obtained in each case. Genomic DNA was extracted using QlAamp DNA kit (Qiagene, Valencia, CA, USA) and subjected to EpiTect Bisulfite kit (Qiagene) according to the manufacture's protocol. In brief, 2 μg of genomic DNA was subjected to a denature/incubation repeat cycle in sodium bisulfite solution.

### Quantitative real time methylation specific PCR (qMSP)

Bisulphite converted DNA was subjected to real time qMSP using ABI StepOne real time PCR system (Applied Biosystems, Foster City, CA) as previously described [30] with slight modification. In Brief, each reaction contained 12.5 μL of 2 × SYBR green PCR mix (Toyobo, Japan), 160 nM of each primer and 4 μl of bisulphite modified DNA in a total volume of 25 μl at 95°C for 10 min, 40 cycles of 95°C for 15 sec, 67°C for 30 sec, and 72°C for 30 sec. Primers targeting the *IGFBP3 *promoter region were shown in Table 4. *β-actin *(ACTB) was used to normalize for input DNA. A region of ACTB devoid of any CpG dinucleotide was amplified using the following primer sequences: forward, 5' TGGTGATGGAGGAGGTTTAGTAAGT and reverse, 5' AACCAATAAAACCTACTCCTCCCTTAA. The amount of methylated IGFBP3 and ACTB were determined by the threshold cycle number (Ct) for each sample against a standard curve generated by *Sss*I-treated DNA (Millipore, Billerica, MA)-MSP cloned fragment. The sequence of the fragment was confirmed by sequencing reaction. The performance of the standard curve was shown in additional file 1, Figure S3. The percentage of IGFBP3 methylation was calculated as the IGFBP3:ACTB ratio of a sample divided by the same ratio of *SssI*-treated sperm DNA (Millipore, Billerica, MA) and multiplying by 100

**Table 4:** Primer sequences for *IGFBP-3 *analysis.

		Annealing °C (Cycle)	Size (bp)
**Quantitative real time methylation-specific PCR**			
*IGFBP3*	F: 5'- AGGTGATTCGGGTTTCGGGC -3' R: 5'- GACCCGAACGCGCCG -3'	60 (40)	223
**Methylation-specific PCR**			
*IGFBP3*_MSP	F: 5'-TCGGGTATATTTTGGTTTTTGTAG-3' R: 5'-AAACATATAAAATCCAAACAAAAA-3'	55 (30)	351
*IGFBP3*_M (methythlated)	F: 5'CGAAGTACGGGTTTCGTAGTCG-3' R: 5'-CGAC CCGAACGCGCCGACC-3'	66 (40)	129
*IGFBP3*_U (unmethylated)	F: 5'-TTGGTTGTTTAGGGTGAAGTATGGGT-3' R: 5'-CACCCAACCACAATACTCACATC-3'	64 (40)	158
**Bisulphate-PCR**			
*IGFBP3*	F: 5'-TTTGAGAGTGGAAGGGGTAAGGG-3' R: 5'-CCCACTACATAACACCTACAACC-3'	53 (40)	512
**Mutagenesis**			
Wild type *IGFBP3*	F: 5'-GGGCACACCTTGGTTCTTGTAG-3' R: 5'-TTCCTGCCTGGATTCCACAGCT-3'	52 (40)	316
Mutant (M12) *IGFBP3*	F: 5'-ACAAGGTGACCCGGGCTCAGGGAGTGAGCACGAGGAGCAGGT-3' R: 5'-ACCTGCTCCTCGTGCTCACTCCCTGAGCCCGGGTCACCTTGT-3'	55 (12)	
Mutant (M34) *IGFBP3*	F: 5'-GCACGAGGAGACGGTGCCAGGGAGAGTCTCAAGCTCCACGCC-3' R: 5'-GGCGTGCAGCTTGAGACTCTCCCTGGCACCTGCTCCTCGTGC-3'	55 (12)	

### Methylation-specific PCR (MSP)

Primers for methylated and unmethylaled region of *IGFBP-3 *promoter, as previously reported [31], are listed in Table 3. The schematic diagram showing the locations of primers used are shown in Figure 1a. Twenty μL of reaction volume containing one-twentieth of the modified DNA, 4 deoxynucleoside triphosphates, PCR primers (-MSP) and HotStar TaqDNA MasterMix (Qiagen, Valencia, CA) were set for first PCR. The PCR product was then used as DNA template for a second methylated (-M) or unmethylated (-U) IGFBP-3 PCR reaction. Results were resolved by electrophoresis using 2% agarose gels. Cell line A549 treated with *Sss*I was used as a positive methylated control.

### Bisulfite-PCR and sequencing (BSP)

The -372 ~+140 bp from the first exon of IGFBP-3 promoter region was amplified from one-twentieth of the modified DNA by PCR using HotStarTaq DNA Master Mix Kit (Qiagene, Valencia, CA). The primer sequences and PCR conditions are shown in Table 4. The pGEM-T Easy Vector (Promega Corp., Madison, WI, USA) was used for TA cloning and high-efficiency competent cells DH5α (Yeastern, Taipei, Taiwan) was used for transformation. Colonies were selected and rechecked by electrophoresis. The recovered plasmids were sequenced at the Sequencing Core Facility of the NTU College of Medicine. Cytosines in CpG dinucleotides that remained unconverted after bisulfite treatment were considered as methylated.

### Western blotting

Twenty-fold concentrated conditioned media, collected from cells cultured in FCS-free DMEM media for 24 hours, were subjected to 8% SDS-PAGE (Millipore, Bedford, MA, USA) and then transferred to membranes (Schleicher & Schuell, Germany) using MilliBlot-SDS semi-dry electroblotting system (Millipore). After blocking, the membrane were probed with antibodies against IGFBP-3 (Research Reagents, Texas, USA). Reactions were amplified by biotinylated second antibody, followed by streptavidin-HRP. Results were exposed using ECL system.

### cDNA preparation and quantitative real time reverse-transcriptase PCR (QRT-PCR)

Total RNA was extracted using TRIzol reagent (Invitrogen, Carlsbad, CA, USA) and cDNA was prepared using SuperScript III First-strand Synthesis of the Oligo (dT) primer system (Invitrogen). The cDNA samples were subjected to QRT-PCR as described previously [5] using 7700 Sequence Detector (Applied Biosystems, Foster City, CA, USA) and SYBR green Master Mix Kit (Alppied Biosystems). Primer sequences for IGFBP-3 were forward: 5' TGTGGCCATGACTGAGGAAA, reverse: 5' TGCCAGACCTTCTTGGGTTT; and GAPDH were forward: 5' TGGTATCGTGGAAGGACTCA, reverse: 5' AGTGGGTGTCGCTGTTGAAG. Each target gene was normalized to GAPDH mRNA expression for comparison.

### Construction of wild and mutant expression vectors

The promoter region of IGFBP-3 (from -253 ~+61) and the site-directed mutagenesis constructs at 4 different mutant sites (C to A at -210, -206, -183 and -179) were generated by PCR amplification with primers and PCR conditions as shown in Table 4. Wild type pGL3-IGFBP-3 and the mutant constructs were generated by subcloning of the PCR fragments to pGL3-Basic plasmid (Promega Corp.). The mutant constructs were named as Mut A (mutation at -210 and -206), Mut B (mutation at -183 and -179) and Mut A+B (mutant at all of the four sites), respectively, as shown in Figure 3c. All constructs were confirmed by DNA sequencing.

### Transient transfection and luciferase assays

Cells were transiently transfected using Arrest-In transfection reagents (Open Biosystems, Huntsville, USA). Cells at a density of 1 × 10^5^/well were seeded in 6-well plate. Transfection was performed using 2 μg of plasmid DNA in serum free medium for 6 hours. Cells were then incubated in serum containing medium for another 48 hours. After washing twice with PBS, cells were collected for luciferase assays (Promega Corp.) using a dual luciferase reporter assay system according to the manufacture's instructions. The relative luciferase activity was normalized against renilla activity by co-transfection with 1 μg of pRL-TK (Promega Corp.).

### Statistical analysis

The cutoff value of *IGFBP-3 *methylation as a threshold of hypermethylation was identified using ROC curve and AUC. The expressions of IGFBP-3 and p53, and *IGFBP-3 *methylation status were compared among patients with different clinical and pathologic features using *t *test and χ^2 ^test. Associations among IGFBP-3 expression, p53 overexpression and *IGFBP-3 *promoter methylation with progression-free survival (PFS) and overall survival (OS) rates were assessed by Cox-regression analysis. Kaplan-Meier analysis with logrank test was used to estimate survival probabilities and to compare survival distributions categorized by IGFBP-3 expression, *IGFBP-3 *promoter methylation and p53 overexpression. Statistical analysis was carried out using Statistical Analysis System (SAS) version 8.0 (SAS Institute, Cary, NC). Probability values less than 0.05 were regarded as significant.

## Competing interests

The authors declare that they have no competing interests.

## Authors' contributions

PLT designed and directed the study, analyzed and interpreted all the data, and drafted the manuscript. PLT also performed IHC and the results were interpreted by PLT and CTL. CWL carried out the MSP, BSP, Western blot analysis, QRT-PCR and site directed mutagenesis assays. MWC and HWY carried out qMSP assays. CTL and SCH participated in the study coordination and helped draft the manuscript. All authors read and approved the manuscript.

## Supplementary Material

Additional file 1 Table S1 and Figure S1, S2, S3. Click here for file
