# Supplementary material for: Promoter methylation of IGFBP-3 and p53 expression in ovarian endometrioid carcinoma
Source: Mol Cancer. 2009 Dec 11;8:120. doi: 10.1186/1476-4598-8-120 (PMC2799391; doi:10.1186/1476-4598-8-120)
Supplement: Additional file 1 — Table S1 and Figure S1, S2, S3. [file 1476-4598-8-120-S1.DOC]

**Table S1: IGFBP-3 and p53 expression and the cliniocopathological features in OEC patients.**

|  | Patients included for qMSP  (n = 40) | Patients excluded  from qMSP  (n = 20) | *p* value |
| --- | --- | --- | --- |
| Age (mean ± SD) | 51.7 ± 13.0 | 51.3 ± 11.4 | 0.92 |
| Stage  I  II  III  IV | 19  5  15  1 | 9  2  9  0 | 1.00 |
| Grade  1  2  3 | 15  14  11 | 4  9  6 | 0.44 |
| Low IGFBP-3 | 14 (35.0 %) | 10 (50.5 %) | 0.40 |
| p53 overexpression | 14 (35.0 %) | 7 (35.0 %) | 0.77 |
| Patients with recurrent tumor | 16 (40.0 %) | 6 (30.0 %) | 0.64 |
| Patients expired | 13 (32.5 %) | 5 (25.0 %) | 0.77 |


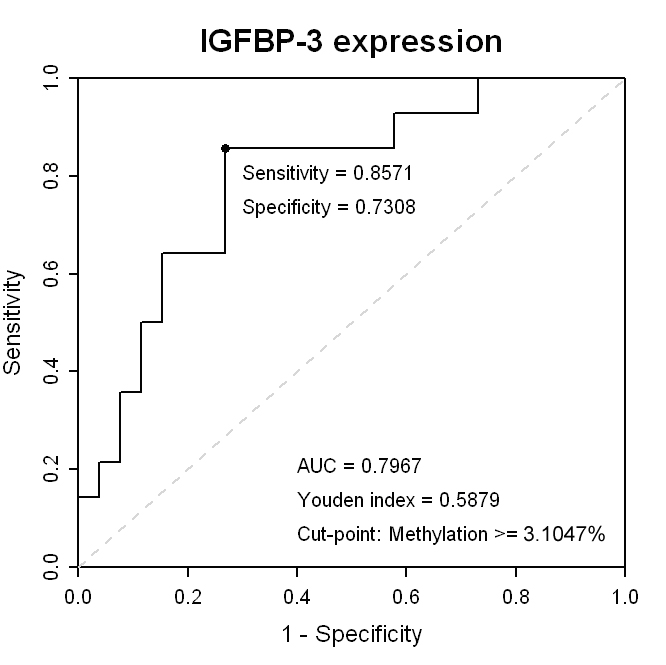


**Figure S1.**

**Receiver-operator characteristic curve of the *IGFBP-3* methylation based on IGFBP-3 expression.**


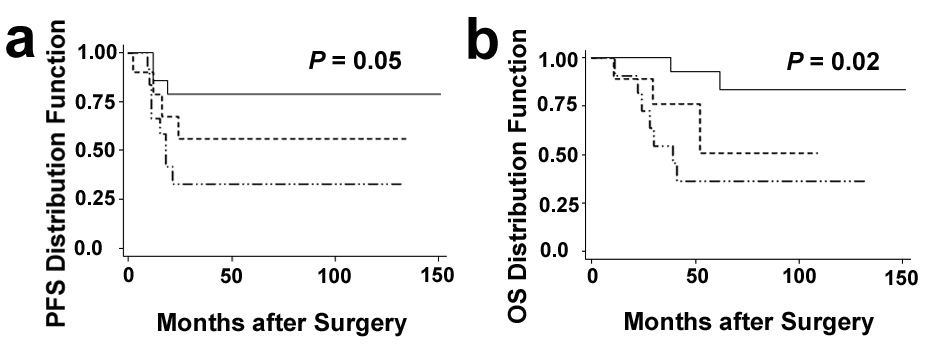


**Figure S2.**

**Kaplan Meier survival estimate of progression-free survival (PFS) (a) and overall survival (OS) (b) for OEC patients with normal or overexpressed p53, according to IGFBP-3 expressions and *IGFBP-3* promoter methylation status.** Logrank test was used to compare survival distributions by IGFBP-3 expressions and *IGFBP-3* methylation status. indicates patients with high IGFBP-3 and *IGFBP-3* promoter methylation ≤ 3%; indicates patients with unparallel IGFBP-3 and *IGFBP-3* promoter methylation, i.e. either high IGFBP-3 and *IGFBP-3* promoter methylation > 3% or low IGFBP-3 and *IGFBP-3* promoter methylation ≤ 3%; indicates patients with low IGFBP-3 and *IGFBP-3* promoter methylation > 3%.


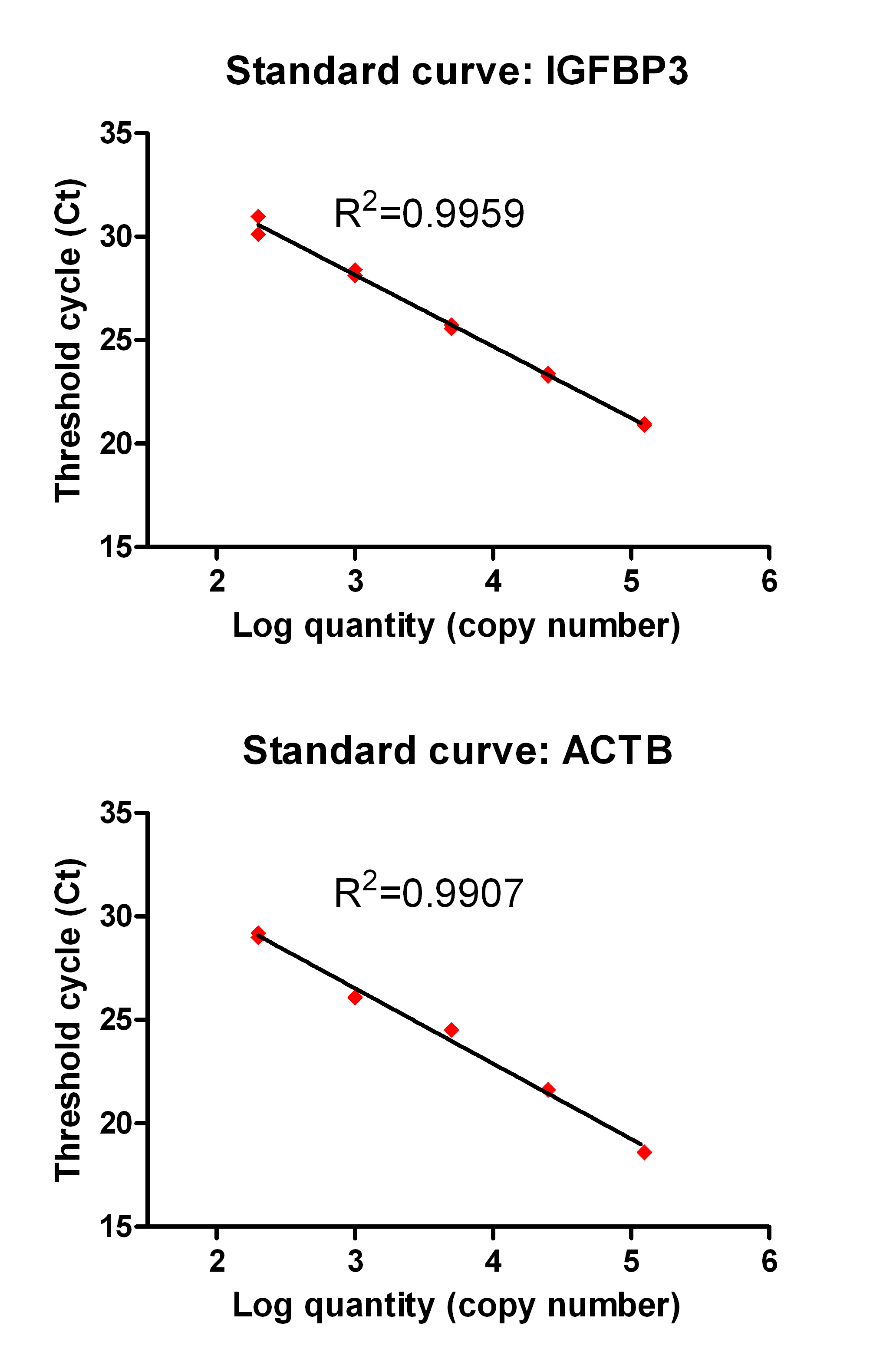


**Figure S3.**

**The standard curve of IGFBP3 (upper) and ACTB (lower) in the qMSP reaction.** To generate a standard curve, a commercially available *Sss*I-treated DNA was first used to perform MSP reaction using specific primer. The MSP fragment was then cloned into pCRII cloning vector using TOPO-TA cloning kit. After confirmation by sequencing, the circular plasmid was linearized by BamHI and different copy number (40, 200, 1000, 5000, and 25000) of the linearized plasmid DNA was used as template to generate the standard curve for qMSP reaction.
